# Influence of strangers' vocal attractiveness on adolescents' epistemic trust: moderation effect of analytical thinking

**DOI:** 10.3389/fpsyg.2026.1700278

**Published:** 2026-02-16

**Authors:** Cui Zhu, Yiran Zhang, Zan Liu, Dongjie Xie, Cheng Yang, Yanjie Su

**Affiliations:** 1 School of Design and Fashion, Zhejiang University of Science and Technology, Hangzhou, China; 2 Huawei Cloud, Hangzhou, China; 3 School of Education and Music, Sanming University, Sanming, China; 4 Guangdong Institute of Public Administration, Guangzhou, China; 5 School of Psychological and Cognitive Sciences, Peking University, Beijing, China

**Keywords:** adolescence, analytical thinking, cognitive bias, epistemic trust, vocal attractiveness

## Abstract

Epistemic trust is closely linked to the cognitive process of information containing knowledge toward the adaptation to complex social environments, and can be influenced by cognitive biases from external traits such as the voice of a stranger. This influence may be moderated by factors related to the individual's thinking style and the voice's emotional content. Considering that adolescence is an important stage in the social development of individuals, we investigate the influence of strangers' vocal attractiveness on adolescents' epistemic trust, as well as its moderators, via two studies based on trustworthiness ratings of technology news summaries contained in audio messages. Experimental results suggest that vocal attractiveness is a crucial cue promoting epistemic trust when adolescents make relevant decisions during interactions with strangers. In addition, successfully activating individuals' analytical thinking has been found to reduce this influence.

## 1 Introduction

Trust, acknowledged as integral to human interaction, plays a crucial role in fostering positive interpersonal relationships and enabling cooperation across various contexts (Berscheid, 1994). Within the domain of knowledge dissemination, epistemic trust emerges as a developmental and relational process that facilitates openness to communication and social learning, particularly during adolescence (Fonagy and Allison, 2014; Fonagy et al., 2015). Grounded in mentalization theory, epistemic trust represents the ability to discern the reliability of knowledge sources while balancing epistemic vigilance (Fonagy et al., 2019). This dynamic process is essential for adaptive social-cognitive maturation, as it enables individuals to selectively assimilate new information within relational contexts (Parolin et al., 2023). Empirical studies highlight its associations with mentalization capacity and emotion regulation, underscoring its role in adolescents' psychosocial development (Locati et al., 2023; Milesi et al., 2024).

While previous studies have underscored the significance of epistemic trust, particularly in adolescents (Jaffrani et al., 2020), empirical investigations remain notably limited. Furthermore, although researchers have attempted to explore the determinants of epistemic trust (Imhoff and Lamberty, 2018), the influence of physical attractiveness, which is a significant social cue, remains unexplored. Considering vocal attractiveness, defined as the desirability of vocalizations to potential mates (Hill and Puts, 2016), is a critical component of physical attractiveness, this study involves two empirical studies examining its influence on adolescents' epistemic trust. By elucidating how physical attractiveness, as manifested in voice, intersects with adolescents' trust decision-making processes, we aim to deepen the understanding of trust dynamics while offering theoretical insights to enhance adolescents' knowledge acquisition.

## 2 Related work

### 2.1 Influence of vocal attractiveness on epistemic trust

Human voices contain considerable clues that can rapidly shape impressions of personality in others (Yovel and Belin, 2013). The tendency of individuals to judge one's trustworthiness based on vocal attractiveness upon the initial exposure can be explained as that specific brain regions (e.g., amygdala), as a common neural substrate in affective voice processing, are evolutionarily significant regarding long-term information filtering (Bzdok et al., 2010). Hence, vocal attractiveness is likely to be associated with decision making on trust (Shang and Liu, 2022).

Previous studies on the influence of voice on trust depended on the manipulation of pitch and speech rate. For example, Torre (2017) found that both high pitch and slow speech increased the likelihood of being invested in investment games. Although vocal traits and vocal attractiveness may receive consistent judgements and function closely on impressions, it is still necessary to validate if the latter possesses a similar influence on trust since its measurement differs from that of the former, given its conceptual definition (Hill and Puts, 2016).

In terms of epistemic trust, if the influence of vocal attractiveness existed, it could be interpreted as a *halo effect* triggered by vocal attractiveness, which is an important component of physical attractiveness. Since halo effect is a cognitive bias through which positive evaluations of one attribute (e.g., attractiveness) unconsciously influence judgments of unrelated traits (e.g., competence or trustworthiness), it has often been adopted to explain stereotypical impressions associated with physical attractiveness (Han et al., 2018). On this basis, we propose the following hypothesis:

- **H1**: *Adolescents tend to trust the information conveyed by vocally attractive strangers*.

### 2.2 Moderation effects of analytical thinking on cognitive biases

Cognitive biases, including the halo effect, are caused by the dependence on intuition in decision-making and reasoning. According to information processing theory, individuals' cognitive abilities continue to grow during adolescence, which is particularly evident regarding information acquisition, utilization, and storage, as well as strategies for dealing with new situations (Wyer, 2012). Cognitive biases may stem from two different modes of information processing: *intuitive thinking* and *analytical thinking*, between which the conflict has been known as the dual-process theory (De Neys, 2012).

Specifically, human brains adopt two cognitive systems: System 1 operates autonomously and in parallel with high speed and large capacity, and does not require working memory, whereas System 2 operates cautiously and in a serial manner with strong analytical capabilities, and relies on working memory and consumes more resources (Evans and Stanovich, 2013). Analytical thinking, which represents the processing of System 2, is regarded as the cornerstone of rational thinking and is believed to be an important factor moderating individuals' cognitive biases. Once analytical thinking is activated, individuals tend to make more rational judgments by exploiting known information and thus correct cognitive biases resulting from intuitive thinking.

Therefore, analytical thinking may be a cognitive moderator of vocal attractiveness's influence on epistemic trust. Previous studies have shown that decisions made by participants who unconsciously leaned toward analytical thinking outperformed those made by participants consciously biased toward intuitive thinking (Zhou et al., 2015). Besides, analytical thinking exhibits significant differences in organizing and representing information from intuitive thinking. For example, Wen et al. (2020) validated that participants in an analytical thinking state exhibited weaker halo effects caused by the central traits of objects (e.g., warmth and coldness). On this basis, we propose the following hypothesis:

- **H2**: *Activating the analytical thinking of adolescents can reduce the influence described in H1*.

## 3 Study 1

Based on an audio message rating experiment, Study 1 aimed to investigate how stranger vocal attractiveness would influence adolescent epistemic trust. Adolescent participants were recruited to listen to audio messages recorded by strangers, each of which conveyed a technology news summary, and then rate these messages regarding the information trustworthiness of the corresponding summaries.

The operationalization of epistemic trust through information trustworthiness rating effectively captures the core dimensions of cognitive openness and relevance appraisal unique to epistemic trust (Milesi et al., 2024). Unlike general credibility assessments, this measure requires participants to evaluate not just factual accuracy, but also personal applicability, mirroring key items (e.g., trusting information more when it feels personally resonant) from the existing questionnaire on epistemic trust (Campbell et al., 2021). By manipulating vocal attractiveness, this measure assesses context-sensitive openness to knowledge transmission (Campbell et al., 2021)., distinguishing adaptive epistemic trust from indiscriminate credulity or rigid mistrust. Its behavioral nature aligns with the definition of epistemic trust while avoiding the limitations of explicit self-reports (Milesi et al., 2024).

### 3.1 Participants

In this study, 243 students from a primary school, a secondary school, and a university in China (152 women, 91 men; *M*_*age*_ = 17.34 years, *SD*_*age*_ = 2.63 years, from 12 to 22 years) were recruited through on-campus advertisements. Those participants from the university were individually paid 50 Chinese yuan (circa 7 US dollars), and each of the rest was given a gift notebook for their participation. All the participants were blinded to the experimental purpose and completed the experiment. None of them were excluded.

### 3.2 Materials

The materials comprised a set of audio messages conveying the selected technology news summaries. At the beginning, we recruited 66 readers (33 women, 33 men; *M*_*age*_ = 28.72 years, *SD*_*age*_ = 2.32 years, from 26 to 31 years) in China, who were strangers to all the participants, through an instant messenger-based online advertisement. Each reader was asked to record an example audio message conveying a simple fact (i.e., “The year 2024 is a leap year with three hundred and sixty-six days.”) at a normal speed and a calm tone using standard Mandarin Chinese. Since individuals have been found to rate voice attractiveness similarly in adolescence and adulthood (Saxton et al., 2009), we invited 30 independent raters from a university in China (15 women, 15 men; *M*_*age*_ = 20.15 years, *SD*_*age*_ = 1.23 years, from 18 to 22 years) through an on-campus advertisement to rate the readers regarding vocal attractiveness. Specifically, each rater listened to an example audio message followed by a question (i.e., “Do you agree that the reader's voice is attractive?”) and completed the ratings using a 7-point Likert scale from 1 (i.e., strongly disagree) to 7 (i.e., strongly agree) on an online survey platform. For each rater, 66 trials were conducted, and all the messages were provided in a random order. The inter-rater reliability was high with a Cronbach's α of 0.94. Using the reader's average rating (*M* = 4.16, *SD* = 0.79) as a *vocal attractiveness score* (VAS), we regarded the top 3 readers with *high vocal attractiveness* and those bottom 3 with *low vocal attractiveness* by the reader's gender. These 12 readers (6 women) were invited to record the audio messages required in the following experiments.

Afterwards, we selected 12 technology news reports from the website of *Science* and produced corresponding one-sentence summaries after translation and summarization. To rate the technology news summaries regarding information trustworthiness, we invited 45 independent raters (22 women, 23 men; *M*_*age*_ = 31.60 years, *SD*_*age*_ = 5.70 years, from 22 to 40 years) in China through an instant messager-based online advertisement. Specifically, each rater viewed a summary followed by a question (i.e., “Do you agree that the information in this technology news summary is trustworthy?”) and completed the rating using a 7-point Likert scale from 1 (i.e., strongly disagree) to 7 (i.e., strongly agree) on an online survey platform. For each rater, 12 trials were conducted, and all the summaries were displayed in a random order. The inter-rater reliability was high with a Cronbach's α of 0.71. One-sample t-tests showed that summary-wise average rating (*M* = 4.70, *SD* = 0.33) was overall larger than 4 (i.e., neutral), *t*_(11)_ = 6.81, *p < *0.001, and less than 5 (i.e., somewhat agree), *t*_(11)_ = −3.34, *p* = 0.007, demonstrating that all the summaries were regarded neutral. Therefore, these summaries were used for recording the audio messages required in the following experiments, and summary-wise average rating was adopted as a *baseline trustworthiness score* (BTS) for each summary (see Table 1).

**Table 1:** Selected technology news summaries prior (in English) and corresponding baseline trustworthiness scores (BTS).

**ID**	**Technology news summary**	**BTS**
1	Lifting giants in gravity batteries can be used to store renewable energy.	4.06
2	Magnetars release powerful flashes of light when being torn apart in a starquake.	4.79
3	The lifespan of social insects varies markedly depending on their division of labor.	5.15
4	Radar satellites can detect insignificant rises and falls in the Earth's surface.	4.53
5	Future detectors can acquire low-frequency gravitational waves from black holes.	4.49
6	A worm without eyes relies on stress genes to capture blue and yellow light.	4.85
7	Fin whales' songs can serve crust imaging and undersea exploration.	4.81
8	Small mammals living in groups can identify themselves by emitting faint chirps.	4.98
9	Animal observations in the Arctic Circle benefit the detection of climate change effects.	5.17
10	Sediment delivery to sinking areas via rivers increases the size of a coast.	4.43
11	Electrical coupling between sound sources produces distinctive voices.	4.43
12	Signals from next-generation cell phones can interfere with weather prediction.	4.04

Each of the invited 12 readers was asked to record audio messages conveying the selected 12 technology news summaries at a normal speed and a calm tone using standard Mandarin Chinese. Afterward, we wrote a Python program to randomly assign an audio message from the 12 recorded samples to each technology news summary, while each reader only corresponded to one summary. The selected 12 audio messages were used as materials in the following experiments.

### 3.3 Procedure

The experiment adopted a 2 × 2 within-subject design (reader's vocal attractiveness: high and low; reader's gender: female and male), leading to 4 conditions, each of which comprised 3 trials. Each participant completed the experiment on a desktop computer.

At the beginning, the monitor displayed a welcome message and an experiment introduction, followed by showing a *Play* button at the center. The participant was instructed to click the button and listen to the audio message being played. Afterwards, a question was shown below the *Play* button (i.e., “Generally speaking, does the information sound trustworthy?”) for the participant to answer using a 7-point Likert scale (1: strongly untrustworthy; 2: untrustworthy; 3: somewhat untrustworthy; 4: neutral; 5: somewhat trustworthy; 6: trustworthy; and 7: strongly trustworthy), which concluded a trial. The experiment ended after the participant accomplished all the 12 trials, which were provided in a random order. Any reader or summary involved only once in the experiment. Following the experiment, the participant reported demographic variables, including date of birth and gender.

### 3.4 Data analysis and results

Experimental results were analyzed via *linear mixed model* (LMM) using Jamovi. For each trial, we adopted the difference between a participant's rating for the audio message and the BTS for the corresponding technology news summary as an *trustworthiness perception score* (TPS). The higher the TPS is, the more trustworthy the summary's information sounded to the participant.

To investigate how readers' vocal attractiveness influences participants' perception of the summary's information trustworthiness, we conducted an LMM analysis with the summary's TPS as the dependent variable and the reader's VAS as the main independent variable with a fixed effect. Participants were treated as random effects. As shown in Table 2, the main effect of VAS was significant, *b* = 0.09, *t* = 4.21, *p* < 0.001, suggesting a positive influence on information trustworthiness toward those technology news summaries expressed by vocally attractive readers.

**Table 2:** Results of the LMM analysis on the relationship between summary's trustworthiness perception score (TPS) and reader's vocal attractiveness score (VAS).

	***b***	***SE***	***df***	***t***	***p***	**95% CI**
Intercept	–0.06	0.05	242	–1.24	0.217	
VAS	0.09	0.02	2672	4.21	< 0.001	[0.05, 0.13]

We performed 100 simulations of the above LMM on the experimental data using the *powerSim* function of *simr*, which indicated the LMM's statistical power of 0.85 and VAS's effect size of 0.08.

## 4 Study 2

The goal of Study 2 was to examine how analytical thinking would moderate the influence suggested by the results of Study 1. Prior to the same audio message rating experiment, participants in the experimental group engaged in a task on activating their analytical thinking.

### 4.1 Participants

In this study, 258 students from a primary school, a secondary school, and a university in China (153 women, 105 men; *M*_*age*_ = 16.60 years, *SD*_*age*_ = 3.18 years, from 12 to 22 years) were recruited through on-campus advertisements. Those participants from the university were individually paid 50 Chinese yuan (circa 7 US dollars), and each of the rest was given a gift notebook for their participation.

We assigned the participants to two groups: an experimental group (*N* = 132; 79 women, 53 men; *M*_*age*_ = 16.40 years, *SD*_*age*_ = 3.14 years, from 12 to 22 years) and a control group (*N* = 125; 74 women, 51 men; *M*_*age*_ = 16.90 years, *SD*_*age*_ = 3.20 years, from 12 to 22 years). Participants were blinded to the experimental purpose and completed the experiment. None of them were excluded.

### 4.2 Procedure

The experiment adopted a 2 × 2 × 2 mixed design (reader's vocal attractiveness: high and low; reader's gender: female and male; group: experimental and control), leading to 8 conditions. Each participant completed the experiment on a desktop computer.

Following a welcome message and an experiment introduction displayed by the monitor, the participant was initially asked to perform a group-specific task. Specifically, one in the experimental group answered 36 non-verbal intelligence measurement questions, which were selected from the Raven's progressive matrices test; one in the control group viewed 36 webpages, each of which showed at the center 4 scenic photographs for non-commercial use from the Internet and below a question irrelevant to analytical thinking (i.e., “Which photograph do you like the best?”). Afterwards, the participant accomplished the same 12 trials as in Study 1, followed by answering the 3 questions from the cognitive reflection test (CRT) (i.e., “How much does the ball cost if a bat and a ball cost $1.10 in total and the bat costs $1.00 more than the ball?”; “How long would it take 100 machines to make 100 widgets if it takes 5 machines 5 min to make 5 widgets?”; “How long would it take for the patch of lily pads to cover half of the lake if the patch doubles in size every day and it takes 48 days for the patch to cover the entire lake?”). The participant obtained 1 score if they correctly answered a question, and otherwise 0. Following the experiment, the participant reported demographic variables, including date of birth and gender.

### 4.3 Data analysis and results

Experimental results were analyzed via LMM using Jamovi. For each participant, the average score obtained in the 3 CRT questions was adopted as a metric to evaluate the activation of analytical thinking. To examine its difference, we conducted an independent samples t-test on the average scores for participants in both groups. Results indicated a significant between-group difference regarding the activation of analytical thinking, *t*_(256)_ = 3.86, *p < *0.001, , Cohen's *d* = 0.24; participants in the experimental group were associated with higher average scores (*M* = 0.62, *SD* = 0.03), which suggested that they were in a higher level of analytical thinking, than those in the control group (*M* = 0.46, *SD* = 0.03).

To investigate how analytical thinking moderates the influence of the reader's vocal attractiveness on participants' perception of the summary's information trustworthiness, we conducted an LMM analysis with the summary's TPS as the dependent variable and the reader's VAS as the main independent variable with a fixed effect, while considering the interactions between VAS and *participant's group*. Participants were treated as random effects. As shown in Table 3 the main effect of VAS was significant, *b* = 0.18, *t* = 8.50, *p < *0.001, confirming the existence of the influence.

**Table 3:** Results of the LMM analysis on the relationship between summary's trustworthiness perception score (TPS) and reader's vocal attractiveness score (VAS).

	***b***	***SE***	***df***	***t***	***p***	**95% CI**
Intercept	–0.10	0.04	256	–2.28	0.023	
VAS	0.18	0.02	2836	8.50	< 0.001	[0.14, 0.22]
VAS × Group (experimental)	–0.11	0.04	2836	–2.58	0.010	[–0.19, –0.03]

**Figure 1:**
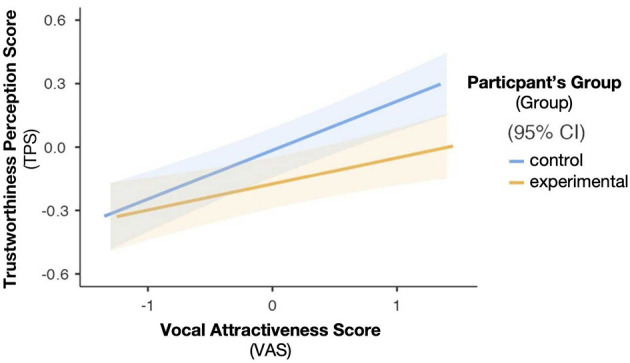
Plotting of the simple slopes analysis on the interaction effect between the reader's vocal attractiveness score (VAS) and the participant's group.

It is noteworthy that a significant interaction effect was found between VAS and participants' group, *b* = −0.11, *t* = −2.58, *p* = 0.010, According to the results of a simple slopes analysis, the influence became less evident in participants in the experimental group, *b* = 0.12, *t* = 4.13, *p < *0.001, than those in the control group, *b* = 0.23, *t* = 7.96, *p < *0.001 (see Figure 1).

We performed 100 simulations of the above LMM on the experimental data using simr's powerSim function, which indicated the LMM's statistical power of 0.90 and VAS's effect size of 0.18.

## 5 Discussion

The experimental results of Study 1 indicated that technology news summaries provided by readers with higher vocal attractiveness received higher trustworthiness ratings from participants. Considering that similar experimental results were obtained in the following study, this phenomenon suggests that the influence of strangers' vocal attractiveness on adolescents' epistemic trust exists as a relatively stable promoting effect, which validates our hypothesis H1. The influence of vocal attractiveness on epistemic trust can be interpreted as the fact that the vocal attractiveness of strangers may work as a heuristic cue for individuals to process relevant information in the default processing mode based on System 1, especially for adolescents who are susceptible to the interferences of environmental factors with their decision-making. This aligns with recent findings on epistemic trust as a critical factor in adolescent psychological functioning, particularly in contexts where heuristic cues dominate decision-making (Milesi et al., 2024).

The experimental results of Study 2 indicated that the influence evaluation scores were significantly lower in the participants with analytical thinking activated. This phenomenon suggests that analytical thinking reduces the promoting effect of strangers' vocal attractiveness on adolescents' epistemic trust, which validates our hypothesis H2. Previous studies have discovered that individuals who support the beliefs in religions, supernatural phenomena, and conspiracy theories are more receptive to relevant biases in syllogistic reasoning tasks due to the lack of analytical thinking (Šrol, 2021). In recent years, the rapid development of social networking services has empowered some organizations to spread fake information based on distorted facts through video clips and microblogs for their own interests, which challenges epistemic trust, especially among adolescents. Regarding this issue, analytical thinking was examined to be negatively correlated with susceptibility to fake information (Bronstein et al., 2019). This can be explained as the capability of analytical thinking to let individuals engage in slow and controlled processing of information, which assists them in establishing a reliable understanding of knowledge and information and reducing cognitive biases. These findings resonate with study by Campbell et al. (2021) and Fonagy et al. (2019), who emphasized the protective role of analytical thinking against epistemic credulity and misinformation. However, while our results suggest modulation by analytical thinking, causal developmental inferences remain limited due to the cross-sectional design, echoing cautionary notes in interpreting epistemic trust dynamics (Parolin et al., 2023).

Our studies in this study targeted the entire adolescence, spanning a wide range of ages. However, this design can hardly explore the continuous development of the influence and may involve interaction with the era. Future studies could employ longitudinal design and sequential design for systematic and regular follow-ups of participants at different stages of adolescence, providing a thorough characterization of how strangers' vocal attractiveness influences adolescents' epistemic trust from a perspective of development, following the recommendations by Locati et al. (2023), underscoring the need for developmental perspectives in studying epistemic trust.

Besides, vocal attractiveness in our studies was referred to as the extent to which a voice stimulus induces a positive and pleasurable emotional experience and drives others to approach it, which is relatively broad. Previous studies found that adult participants with reading disabilities showed significantly lower recognition of native voices compared to normal readers, but this difference was not significant when recognizing non-native voices. It suggests that individuals' language familiarity can influence their voice recognition and, moreover, language-related factors may moderate the influence of vocal attractiveness. Therefore, refining the definition of vocal attractiveness and exploring how different components and dimensions of vocal attractiveness influence trust in strangers are worthy of further investigation. In the future, it is worthwhile to explore how different components and dimensions of strangers' voice attractiveness influence epistemic trust, building on recent methodological advances in measuring epistemic trust (Campbell et al., 2021; Milesi et al., 2024).

## 6 Conclusion

Highly attractive voices of strangers are able to promote adolescents' epistemic trust in information containing knowledge. Meanwhile, activating individuals' analytical thinking would reduce the influence.

## Data Availability

The raw data supporting the conclusions of this article will be made available by the authors, without undue reservation.
